# Farkas Certificates and Minimal Witnesses for Probabilistic Reachability Constraints

**DOI:** 10.1007/978-3-030-45190-5_18

**Published:** 2020-03-13

**Authors:** Florian Funke, Simon Jantsch, Christel Baier

**Affiliations:** 8 Johannes Kepler University, Linz, Austria; 9 University of Birmingham, Birmingham, UK; Technische Universität Dresden, Dresden, Germany

## Abstract

This paper introduces *Farkas certificates* for lower and upper bounds on minimal and maximal reachability probabilities in Markov decision processes (MDP), which we derive using an MDP-variant of Farkas’ Lemma. The set of all such certificates is shown to form a polytope whose points correspond to witnessing subsystems of the model and the property. Using this correspondence we can translate the problem of finding minimal witnesses to the problem of finding vertices with a maximal number of zeros. While computing such vertices is computationally hard in general, we derive new heuristics from our formulations that exhibit competitive performance compared to state-of-the-art techniques. As an argument that asymptotically better algorithms cannot be hoped for, we show that the decision version of finding minimal witnesses is ${\text {NP}}$-complete even for acyclic Markov chains.

## 1 Introduction

The goal of program verification is to consolidate the user's trust that a given system works as intended, and if this is not the case, to provide her with useful diagnostic information. Verification tools may, however, contain bugs and so a last grain of insecurity regarding their results always remains. A widely acknowledged approach to overcome this dilemma has been made in the form of *certifying algorithms* [17, 64]. These algorithms provide every result with an accompanying *certificate*, i.e., a token that can be used to verify the result independently and with little ressources. In this way, certificates enable the user (or a third party) to quickly give a mathematically rigorous proof for the correctness of the result *irrespective* of whether the algorithm itself works correctly.

*Counterexamples*, i.e. certificates for the violation of a property, can often be obtained as a byproduct of verification procedures. What constitutes a counterexample is highly context-dependent. Finite executions suffice as counterexamples for safety properties and single, possibly infinite, executions are viable counterexamples for LTL [29]. Tree-like counterexamples have been considered for

* This work was funded by DFG grant 389792660 as part of TRR 248, the Cluster of Excellence EXC 2050/1 (CeTI, project ID 390696704, as part of Germany's Excellence Strategy), DFG-projects BA-1679/11-1 and BA-1679/12-1, and the Research Training Group QuantLA (GRK 1763).

fragments of CTL [28]. For a probabilistic system $\mathcal{M}$ and a linear time property $\phi$, the most prominent notion of counterexample to $\mathrm{Pr}_{\mathcal{M}}(\phi) < \lambda$ is a set of paths satisfying $\phi$ whose probability mass is at least $\lambda$ (see [1] for a survey).

Another notion of counterexample for probabilistic systems $\mathcal{M}$ and properties of the form $\mathrm{Pr}_{\mathcal{M}}(\phi) < \lambda$ are *critical subsystems* [1]. We adopt the reverse perspective and call a subsystem $\mathcal{M}'$ of $\mathcal{M}$ a *witnessing subsystem* for the property $\mathrm{Pr}_{\mathcal{M}}(\phi) \geq \lambda$ if $\mathrm{Pr}_{\mathcal{M}'}(\phi) \geq \lambda$. Small witnessing subsystems offer an insight into what parts of the system are responsible for the satisfaction of the property. Nonetheless, witnessing subsystems can hardly be regarded as viable certificates since verifying $\mathrm{Pr}_{\mathcal{M}'}(\phi) \geq \lambda$ is as hard as checking $\mathrm{Pr}_{\mathcal{M}}(\phi) \geq \lambda$ itself.

In this paper we build a solid bridge between certificates and witnessing subsystems. The systems we consider are modeled as Markov decision processes (MDP), which contain an absorbing goal state representing a desirable outcome. This approach is motivated by the fact that numerous model checking tasks can be reduced to reachability problems [3, 31, 32, 46, 73, 74].

Using Farkas' Lemma, we introduce certificates for bounds on the minimal and maximal probability to reach the goal state. We show that the set of these certificates forms a polytope and we provide a direct translation of a certificate to a witnessing subsystems for lower bounded threshold properties. Thereby, we bridge the gap between an abstract gadget, serving solely as a proof that the result is correct, and a concrete object, containing crucial diagnostic information about *why* the result holds. Moreover, our translation reduces the computation of minimal witnessing subsystems to a purely geometric problem, for which we provide and evaluate new exact and heuristic algorithms.

All omitted proofs can be found in the full version of this paper [42].

**Contributions.**

- Following the concept of certificates in certifying algorithms, we introduce *Farkas certificates* for reachability problems in MDPs (Table 1).
- We give a uniform notion of *witnessing subsystem* (WS) for $\mathbf{Pr}^{\max}_{s_0}(\Diamond \,\mathrm{goal}) \geq \lambda$ and $\mathbf{Pr}^{\min}_{s_0}(\Diamond \,\mathrm{goal}) \geq \lambda$ (Definition 4.1). To the best of our knowledge, witnesses for $\mathbf{Pr}^{\min}_{s_0}(\Diamond \,\mathrm{goal}) \geq \lambda$ have not been considered previously.
- We establish NP-completeness for finding minimal WS even for acyclic discrete time Markov chains (DTMC) (Theorem 4.5).
- Our main result establishes a strong connection between the polytopes of Farkas certificates for $\mathbf{Pr}^{\min}_{s_0}(\Diamond \,\mathrm{goal}) \geq \lambda$ and $\mathbf{Pr}^{\max}_{s_0}(\Diamond \,\mathrm{goal}) \geq \lambda$ and WS of the same property (Theorem 5.4). In particular, one can read off a minimal WS from a vertex of the polytope with a maximal number of zeros (Corollary 5.5).
- From our polytope characterizations we derive two algorithms for computing minimal WS: one based on vertex enumeration and one based on mixed integer linear programming (Section 6). We also introduce a linear programming based heuristic aimed at computing small WS. We evaluate our approach on DTMC and MDP benchmarks, where particularly our heuristics show competitive results compared to state-of-the-art techniques (Section 7).

**Table 1:** Overview of Farkas certificates for reachability properties in MDPs (where $\lesssim \in \{\leq, <\}$ and $\gtrsim \in \{\geq, >\}$).

| Property | Certificate dimension | Certificate condition |
|---|---|---|
| $\mathbf{Pr}^{\min}_{s_0}(\lozenge\, \text{goal}) \gtrsim \lambda$ | $\mathbf{z} \in \mathbb{R}^S$ | $\mathbf{Az} \leq \mathbf{b} \land \mathbf{z}(s_0) \gtrsim \lambda$ |
| $\mathbf{Pr}^{\max}_{s_0}(\lozenge\, \text{goal}) \gtrsim \lambda$ | $\mathbf{y} \in \mathbb{R}^{\mathcal{M}}_{\geq 0}$ | $\mathbf{yA} \leq \delta_{s_0} \land \mathbf{yb} \gtrsim \lambda$ |
| $\mathbf{Pr}^{\min}_{s_0}(\lozenge\, \text{goal}) \lesssim \lambda$ | $\mathbf{y} \in \mathbb{R}^{\mathcal{M}}_{\geq 0}$ | $\mathbf{yA} \geq \delta_{s_0} \land \mathbf{yb} \lesssim \lambda$ |
| $\mathbf{Pr}^{\max}_{s_0}(\lozenge\, \text{goal}) \lesssim \lambda$ | $\mathbf{z} \in \mathbb{R}^S$ | $\mathbf{Az} \geq \mathbf{b} \land \mathbf{z}(s_0) \lesssim \lambda$ |

**Related work.** The fundament of certifying algorithms has been surveyed in [64]. In the context of model checking, the most prominent approach for the certification of a positive result has been to construct a proof of the property in the system [15, 66, 67]. Rank-based certificates for the emptiness of a certain automaton [57] can be used to certify positive model checking results. Model checking MDPs in the presence of multiple objectives has been studied in [37, 39].

Heuristic approaches for computing small witnessing subsystems in DTMCs have been proposed in [5, 7, 49, 51, 52] and implemented in the tool Comics [50]. Witnessing subsystems in MDPs have been considered in [6, 9] and [19], which focuses on succinctly representing witnessing schedulers. The mixed integer linear programming (MILP) formulation of [77, 78] allows for an exact computation of minimal witnessing subsystems for the property $\mathbf{Pr}^{\max}_{s_0}(\lozenge\, \text{goal}) \gtrsim \lambda$. NP-completeness of computing minimal witnessing subsystems in MDPs was shown in [24], but the exact complexity has, to the best of our knowledge, not been determined for DTMCs (the problem was conjectured to be NP-complete in [77]).

Minimal probabilistic counterexamples given as sets of paths can be computed by reframing the problem as a $k$-shortest-path problem [44, 45]. Regular expressions have been considered to succinctly represent the set of paths in [33], and extensions were proposed in [18, 76]. The tool Dipro [4] computes probabilistic counterexamples, and a translation of these to fault trees was given in [56]. Another, learning-based, approach [20] also enumerates paths and produces a witnessing subsystem as a byproduct. But none of these approaches considers state-based minimality. Probabilistic counterexamples can be used to automatically guide iterative and refinement-based model checking techniques [23–25, 27, 48, 53].

Farkas' Lemma is a well-known source of certificates for the (in)feasibility of tasks in combinatorial optimization, operations research, and economics, as presented in the detailed historical account given in [70, pp. 209–226] as well as [62, Chapter 2] and [30, 65, 75]. The lecture notes [71] contain a rich variety of applications of linear programming in general and Farkas' Lemma in particular.

## 2 Preliminaries

**Polyhedra and Farkas' Lemma.** Throughout the article we write the dot product of two vectors $\mathbf{x}, \mathbf{y} \in \mathbb{R}^n$ as $\mathbf{xy}$ or $\mathbf{x} \cdot \mathbf{y}$. A *halfspace* in $\mathbb{R}^n$ is a set

$H = \{\mathbf{v} \in \mathbb{R}^n \mid \mathbf{a} \cdot \mathbf{v} \leq b\}$ for some non-trivial $\mathbf{a} \in \mathbb{R}^n$ and $b \in \mathbb{R}$. A *polyhedron* is the intersection of finitely many halfspaces, and a *polytope* is a bounded polyhedron. A *face* of a polyhedron $P$ is a subset $F \subseteq P$ of the form $F = \{\mathbf{x} \in P \mid \mathbf{a} \cdot \mathbf{x} = \max\{\mathbf{a} \cdot \mathbf{y} \mid \mathbf{y} \in P\}\}$ for some $\mathbf{a} \in \mathbb{R}^n$. A *vertex* of $P$ is a face consisting of only one point.

Farkas' Lemma [38] is part of the fundament of polyhedra theory and linear programming. It provides a natural source of certificates showing the infeasibility of a given system of inequalites, or in other words, the emptiness of the polyhedron described by the system. We will use it in the following version.

**Lemma 2.1 (Farkas' Lemma, cf. [70, Corollary 7.1f on p. 90]).** *Let* $\mathbf{A} \in \mathbb{R}^{m \times n}$ *and* $\mathbf{b} \in \mathbb{R}^m$. *Then there exists* $\mathbf{z} \in \mathbb{R}^n_{\geq 0}$ *with* $\mathbf{A}\mathbf{z} \leq \mathbf{b}$ *if and only if there does* not *exist* $\mathbf{y} \in \mathbb{R}^m_{\geq 0}$ *with* $\mathbf{y}\mathbf{A} \geq 0 \wedge \mathbf{y}\mathbf{b} < 0$.

**Markov decision processes.** A *Markov decision process* (MDP) is a tuple $\mathcal{M} = (S, \mathrm{Act}, \iota, \mathbf{P})$, where $S$ is a finite set of *states*, Act is a finite set of *actions*, $\iota$ is a probability distribution on $S$ called the *initial distribution* of $M$, and $\mathbf{P}\colon S \times \mathrm{Act} \times S \to [0, 1]$ is the *transition probability function* where we require $\sum_{s' \in S} \mathbf{P}(s, \alpha, s') \in \{0, 1\}$ for all $s \in S$ and $\alpha \in \mathrm{Act}$. An action $\alpha$ is *enabled* in state $s \in S$ if $\sum_{s' \in S} \mathbf{P}(s, \alpha, s') = 1$. The set of enabled actions at state $s$ are denoted by $\mathrm{Act}(s)$, and we require $\mathrm{Act}(s) \neq \varnothing$ for all $s \in S$. A *path* in an MDP $\mathcal{M}$ is an infinite sequence $s_0\alpha_0 s_1\alpha_1 ...$ such that $\mathbf{P}(s_i, \alpha_i, s_{i+1}) > 0$ for all $i \geq 0$. A finite path is a finite sequence $\pi = s_0\alpha_0 s_1\alpha_1 ... s_n$ with the same condition for all $0 \leq i \leq n - 1$. In this case, we define $\mathrm{last}(\pi) = s_n$. Denote by $\mathrm{Paths}(\mathcal{M})$ and $\mathrm{Paths_{fin}}(\mathcal{M})$ the set of infinite and finite paths in $\mathcal{M}$.

A *discrete-time Markov chain* (DTMC) is an MDP with a single action which is enabled at every state. If $\mathcal{M}$ is a DTMC, then $\mathrm{Paths}(\mathcal{M})$ carries a probability measure, where the associated $\sigma$-algebra is generated by the cylinder sets $\mathrm{Cyl}(\tau) = \{\pi \in \mathrm{Paths}(\mathcal{M}) \mid \pi \text{ has prefix } \tau\}$ of finite paths $\tau = s_0 s_1 ... s_n$ in $\mathcal{M}$ with probability $\Pr(\mathrm{Cyl}(\tau)) = \iota(s_0) \cdot \prod_{0 \leq i < n} \mathbf{P}(s_i, s_{i+1})$ (fore more details see [13, Section 10.1]). In the following we denote for a finite set $X$ the set of probability distributions on $X$ by $\mathrm{Dist}(X)$. Given $\mu \in \mathrm{Dist}(X)$ let the *support* of $\mu$ be $\mathrm{supp}(\mu) = \{x \in X \mid \mu(x) > 0\}$.

A *deterministic scheduler* is a function $\mathfrak{S}\colon \mathrm{Paths_{fin}}(\mathcal{M}) \to \mathrm{Act}$ such that $\mathfrak{S}(\pi) \in \mathrm{Act}(\mathrm{last}(\pi))$ and a *randomized scheduler* is a function $\mathfrak{S}\colon \mathrm{Paths_{fin}}(\mathcal{M}) \to \mathrm{Dist}(\mathrm{Act})$ such that $\mathrm{supp}(\mathfrak{S}(\pi)) \subseteq \mathrm{Act}(\mathrm{last}(\pi))$ for all $\pi \in \mathrm{Paths_{fin}}(\mathcal{M})$. Given a deterministic (or randomized) scheduler $\mathfrak{S}$, a path $\pi = s_0\alpha_0 s_1\alpha_1 ...$ in $\mathcal{M}$ is an $\mathfrak{S}$*-path* if $\alpha_i = \mathfrak{S}(s_0\alpha_0 ... s_i)$ (or $\alpha_i \in \mathrm{supp}(\mathfrak{S}(s_0\alpha_0 ... s_i))$) for all $i \geq 0$.

We denote by $\Pr^{\mathfrak{S}}$ the probability measure on infinite $\mathfrak{S}$-paths (see [13, Definition 10.92 on page 843] for more details). If we replace $\iota$ with the distribution concentrated on state $s$, then we obtain a probability measure $\Pr^{\mathfrak{S}}_{\mathcal{M},s}$ or short $\Pr^{\mathfrak{S}}_s$ on infinite $\mathfrak{S}$-paths starting in $s$. The scheduler is *memoryless* if $\mathfrak{S}(\pi) = \mathfrak{S}(\mathrm{last}(\pi))$ for all $\pi \in \mathrm{Paths_{fin}}(\mathcal{M})$. We abbreviate memoryless deterministic schedulers as *MD-schedulers* and memoryless randomized schedulers as *MR-schedulers*.

Given a state $t \in S$, we let

$$\mathbf{Pr}_s^{\max}(\lozenge t) = \sup_{\mathfrak{S}} \mathrm{Pr}_s^{\mathfrak{S}}(\lozenge t) \quad \text{and} \quad \mathbf{Pr}_s^{\min}(\lozenge t) = \inf_{\mathfrak{S}} \mathrm{Pr}_s^{\mathfrak{S}}(\lozenge t)$$

denote the maximal and minimal probability to reach $t$ eventually when starting in $s$ and set $\mathbf{Pr}^{\min}(\lozenge t) = (\mathbf{Pr}_s^{\min}(\lozenge t))_{s\in S}$ and $\mathbf{Pr}^{\max}(\lozenge t) = (\mathbf{Pr}_s^{\max}(\lozenge t))_{s\in S}$. The supremum and infimum is indeed attained by an MD-scheduler [13, Lemmata 10.102 and 10.113], thus justifying the superscripts.

**Setting 2.2.** Henceforth we will assume that $\mathcal{M} = (S_{\mathrm{all}}, \mathrm{Act}, \iota, \mathbf{P})$ has a unique initial state $s_0 \in S$ and two distinguished absorbing states fail and goal $\in S_{\mathrm{all}}$, i.e., $\mathbf{P}(\mathrm{goal}, \alpha, s) = 0$ for all $\alpha \in \mathrm{Act}$ and $s \in S_{\mathrm{all}}$ with $s \neq \mathrm{goal}$, and likewise for fail. Here goal represents a desirable outcome of the modeled system and fail an outcome that is to be avoided. We use the notation $S = S_{\mathrm{all}} \setminus \{\mathrm{fail}, \mathrm{goal}\}$, we assume that every state $s \in S$ is reachable from $s_0$. We also assume that under every scheduler fail or goal is reachable from any state, i.e., $\mathbf{Pr}_s^{\min}(\lozenge(\mathrm{goal} \vee \mathrm{fail})) > 0$ for all $s \in S$. If $\mathcal{M}$ does not satisfy this condition from the start, we can apply a standard preprocessing step, which is essentially given by taking the MEC quotient of $\mathcal{M}$, see [2, 3] and also [26]. While it is often easier to verify the condition $\mathbf{Pr}_s^{\min}(\lozenge(\mathrm{goal} \vee \mathrm{fail})) > 0$, it is in fact equivalent to $\mathbf{Pr}_s^{\min}(\lozenge(\mathrm{goal} \vee \mathrm{fail})) = 1$ (see the full version [42]).

Whenever suitable, we denote by $\mathcal{M}$ also the set of enabled state-action pairs, i.e., $\mathcal{M} = \{(s, \alpha) \in S \times \mathrm{Act} \mid \alpha \in \mathrm{Act}(s)\}$. Let $\mathbf{A} \in \mathbb{R}^{\mathcal{M} \times S}$ be defined by

$$\mathbf{A}((s,\alpha),t) = \begin{cases} 1 - \mathbf{P}(s,\alpha,s), & \text{if } s = t \\ -\mathbf{P}(s,\alpha,t), & \text{if } s \neq t \end{cases}$$

We denote by $\mathbf{b} = (\mathbf{b}(s,\alpha))_{(s,\alpha)\in\mathcal{M}} \in \mathbb{R}^{\mathcal{M}}$ with $\mathbf{b}(s,\alpha) = \mathbf{P}(s,\alpha,\mathrm{goal})$ and by $\delta_{s_0}$ the probability distribution that assigns 1 to $s_0$, and 0 to all other states.

The vectors $\mathbf{Pr}^{\min}(\lozenge\,\mathrm{goal})$ and $\mathbf{Pr}^{\max}(\lozenge\,\mathrm{goal})$ can be characterized using the following linear programs. Although this characterization is well-known, we give a proof in the full version [42] due to slight differences with the standard literature.

**Proposition 2.3 (LP characterization, cf. [16, Lemma 8]).** *Let $\mathcal{M}$ be an MDP as in Setting 2.2 and let $\boldsymbol{\delta} \in \mathbb{R}^n_{>0}$. Then the vectors* $\mathbf{Pr}^{\min}(\lozenge\,\mathrm{goal})$ *and* $\mathbf{Pr}^{\max}(\lozenge\,\mathrm{goal})$ *are, respectively, the* unique *solution of the LPs*

$$\max\ \boldsymbol{\delta} \cdot \mathbf{z} \ \text{ s.t. } \ \mathbf{A}\mathbf{z} \leq \mathbf{b} \qquad \text{and} \qquad \min\ \boldsymbol{\delta} \cdot \mathbf{z} \ \text{ s.t. } \ \mathbf{A}\mathbf{z} \geq \mathbf{b}.$$

## 3 Farkas certificates for reachability in MDPs

In this section we establish certificates for the following statements:

(1) All schedulers $\mathfrak{S}$ satisfy $\mathrm{Pr}_{s_0}^{\mathfrak{S}}(\lozenge\,\mathrm{goal}) \gtrsim \lambda$ (i.e., $\mathbf{Pr}_{s_0}^{\min}(\lozenge\,\mathrm{goal}) \gtrsim \lambda$).
(2) Some scheduler $\mathfrak{S}$ satisfies $\mathrm{Pr}_{s_0}^{\mathfrak{S}}(\lozenge\,\mathrm{goal}) \gtrsim \lambda$ (i.e., $\mathbf{Pr}_{s_0}^{\max}(\lozenge\,\mathrm{goal}) \gtrsim \lambda$).
(3) All schedulers $\mathfrak{S}$ satisfy $\mathrm{Pr}_{s_0}^{\mathfrak{S}}(\lozenge\,\mathrm{goal}) \lesssim \lambda$ (i.e., $\mathbf{Pr}_{s_0}^{\max}(\lozenge\,\mathrm{goal}) \lesssim \lambda$).
(4) Some scheduler $\mathfrak{S}$ satisfies $\mathrm{Pr}_{s_0}^{\mathfrak{S}}(\lozenge\,\mathrm{goal}) \lesssim \lambda$ (i.e., $\mathbf{Pr}_{s_0}^{\min}(\lozenge\,\mathrm{goal}) \lesssim \lambda$).

where $\lesssim\ \in \{\leq, <\}$ and $\gtrsim\ \in \{\geq, >\}$. The basis of our construction is the LP characterization of the probabilities above and, crucially, Farkas' Lemma.

**Certificates for universally-quantified statements.** In order to deal with the cases (1) and (3), we need the following lemma proved in the full version [42].

**Lemma 3.1.** *For* $\mathbf{A} \in \mathbb{R}^{\mathcal{M} \times S}, \mathbf{b} \in \mathbb{R}^{\mathcal{M}}$ *as in Setting 2.2, we have for all* $\mathbf{z} \in \mathbb{R}^S$ :

$$\mathbf{A}\mathbf{z} \leq \mathbf{b} \implies \mathbf{z} \leq \mathbf{Pr}^{\min}(\lozenge \,\mathrm{goal})$$
$$\mathbf{A}\mathbf{z} \geq \mathbf{b} \implies \mathbf{z} \geq \mathbf{Pr}^{\max}(\lozenge \,\mathrm{goal})$$

**Corollary 3.2.** *For* $\gtrsim \in \{\geq, >\}$ *and* $\lesssim \in \{\leq, <\}$ *we have*

$$\mathbf{Pr}^{\min}_{s_0}(\lozenge \,\mathrm{goal}) \gtrsim \lambda \iff \exists \mathbf{z} \in \mathbb{R}^S.\ \mathbf{A}\mathbf{z} \leq \mathbf{b} \wedge \mathbf{z}(s_0) \gtrsim \lambda$$
$$\mathbf{Pr}^{\max}_{s_0}(\lozenge \,\mathrm{goal}) \lesssim \lambda \iff \exists \mathbf{z} \in \mathbb{R}^S.\ \mathbf{A}\mathbf{z} \geq \mathbf{b} \wedge \mathbf{z}(s_0) \lesssim \lambda$$

*Proof.* For the direction from left to right, we take $\mathbf{z}$ to be $\mathbf{Pr}^{\min}(\lozenge \,\mathrm{goal})$. The opposite direction follows from Lemma 3.1. □

The right hand sides of Corollary 3.2 provide *certifying* formulations for problems (1) and (3): to check whether the corresponding threshold statement holds, one must merely check whether $\mathbf{z}$ satisfies the inequalities, rather than checking whether $\mathbf{Pr}^{\min/\max}_{s_0}(\lozenge \,\mathrm{goal})$ was computed correctly. If the threshold condition is satisfied, then the vectors $\mathbf{Pr}^{\min/\max}_{s_0}(\lozenge \,\mathrm{goal})$ are also valid certificates.

**Certificates for existentially-quantified statements.** To find certificates for the cases (2) and (4), we calculate:

$$\begin{aligned}
& \mathbf{Pr}^{\min}_{s_0}(\lozenge \,\mathrm{goal}) < \lambda \\
\overset{\text{Cor. 3.2}}{\iff} \quad & \neg \exists \mathbf{z} \in \mathbb{R}^S_{\geq 0}.\ \ \mathbf{A}\mathbf{z} \leq \mathbf{b} \wedge \mathbf{z}(s_0) \geq \lambda \\
\iff \quad & \neg \exists \mathbf{z} \in \mathbb{R}^S_{\geq 0}.\ \ \begin{pmatrix} \mathbf{A} \\ -1\, 0 \ldots 0 \end{pmatrix} \mathbf{z} \leq \begin{pmatrix} \mathbf{b} \\ -\lambda \end{pmatrix} \\
\overset{\text{Lem. 2.1}}{\iff} \quad & \exists \mathbf{y} \in \mathbb{R}^{\mathcal{M}}_{\geq 0}, y^* \geq 0.\ \ (\mathbf{y}, y^*) \begin{pmatrix} \mathbf{A} \\ -1\, 0 \ldots 0 \end{pmatrix} \geq 0 \wedge (\mathbf{y}, y^*) \begin{pmatrix} \mathbf{b} \\ -\lambda \end{pmatrix} < 0 \\
\iff \quad & \exists \mathbf{y} \in \mathbb{R}^{\mathcal{M}}_{\geq 0}.\ \ \mathbf{y}\mathbf{A} \geq \delta_{s_0} \wedge \mathbf{y}\mathbf{b} < \lambda.
\end{aligned}$$

For non-strict inequalities, we apply Farkas' Lemma in the opposite direction:

$$\begin{aligned}
& \mathbf{Pr}^{\min}_{s_0}(\lozenge \,\mathrm{goal}) \leq \lambda \\
\overset{\text{Cor. 3.2}}{\iff} \quad & \neg \exists \mathbf{z} \in \mathbb{R}^S_{\geq 0}.\ \ \mathbf{A}\mathbf{z} \leq \mathbf{b} \wedge \mathbf{z}(s_0) > \lambda \\
\iff \quad & \neg \exists \mathbf{z} \in \mathbb{R}^S_{\geq 0}, z^* \geq 0.\ \ \begin{pmatrix} -\mathbf{A} & \mathbf{b} \end{pmatrix} \begin{pmatrix} \mathbf{z} \\ z^* \end{pmatrix} \geq 0 \wedge \begin{pmatrix} -\delta_{s_0} & \lambda \end{pmatrix} \begin{pmatrix} \mathbf{z} \\ z^* \end{pmatrix} < 0 \\
\overset{\text{Lem. 2.1}}{\iff} \quad & \exists \mathbf{y} \in \mathbb{R}^{\mathcal{M}}_{\geq 0}.\ \ \mathbf{y} \begin{pmatrix} -\mathbf{A} & \mathbf{b} \end{pmatrix} \leq \begin{pmatrix} -\delta_{s_0} & \lambda \end{pmatrix} \\
\iff \quad & \exists \mathbf{y} \in \mathbb{R}^{\mathcal{M}}_{\geq 0}.\ \ \mathbf{y}\mathbf{A} \geq \delta_{s_0} \wedge \mathbf{y}\mathbf{b} \leq \lambda.
\end{aligned}$$

The deductions for $\mathbf{Pr}^{\max}(\lozenge \,\mathrm{goal})$ are analogous, so that we get:

**Proposition 3.3.** *For* $\gtrsim \in \{\geq, >\}$ *and* $\lesssim \in \{\leq, <\}$ *we have*

$$\mathbf{Pr}^{\min}_{s_0}(\Diamond\,\mathrm{goal}) \lesssim \lambda \iff \exists \mathbf{y} \in \mathbb{R}^{\mathcal{M}}_{\geq 0}.\ \mathbf{yA} \geq \delta_{s_0} \wedge \mathbf{yb} \lesssim \lambda$$
$$\mathbf{Pr}^{\max}_{s_0}(\Diamond\,\mathrm{goal}) \gtrsim \lambda \iff \exists \mathbf{y} \in \mathbb{R}^{\mathcal{M}}_{\geq 0}.\ \mathbf{yA} \leq \delta_{s_0} \wedge \mathbf{yb} \gtrsim \lambda$$

Together, Corollary 3.2 and Proposition 3.3 give us all certificate conditions of Table 1.

## 4 Minimal witnesses for reachability in MDPs

In this section we consider the following problem: Given an MDP $\mathcal{M}$ that satisfies the property $\mathbf{Pr}^{\min}_{\mathcal{M},s_0}(\Diamond\,\mathrm{goal}) \geq \lambda$ (or $\mathbf{Pr}^{\max}_{\mathcal{M},s_0}(\Diamond\,\mathrm{goal}) \geq \lambda$), find a small subsystem $\mathcal{M}'$ of $\mathcal{M}$ that still satisfies these thresholds. Such a subsystem is a witness to the satisfaction of the property in $\mathcal{M}$. We first define subsystems and consider different measures of size which we show to be equivalent. Then we deal with the question of finding minimal witnessing subsystems.

**Subsystems, witnesses and notions of minimality.** Our definition of subsystem is essentially the same to the definition in [77, 78] that was used for witnessing subsystems of $\mathbf{Pr}^{\max}_{\mathcal{M},s_0}(\Diamond\,\mathrm{goal}) \gtrsim \lambda$. From now on we restrict our attention to properties of the form $\mathbf{Pr}^{\min/\max}_{\mathcal{M},s_0}(\Diamond\,\mathrm{goal}) \gtrsim \lambda$. One can deal with upper bounds by exchanging the roles of fail and goal and invoking the equality $\mathbf{Pr}^{\min}_{\mathcal{M},s_0}(\Diamond\,\mathrm{goal}) = 1 - \mathbf{Pr}^{\max}_{\mathcal{M},s_0}(\Diamond\,\mathrm{fail})$, which holds by the conditions of Setting 2.2.

Intuitively, a subsystem $\mathcal{M}'$ of $\mathcal{M}$ contains a subset of states of $\mathcal{M}$, and a transition of $\mathcal{M}$ originating in a state of $\mathcal{M}'$ remains unchanged in $\mathcal{M}'$ or is redirected to fail (instead of explicitely redirecting to fail, sub-stochastic distributions are used in [77, 78] with the same effect).

**Definition 4.1 (Subsystem and witness).** *Let* $\mathcal{M} = (S_{\mathrm{all}}, \mathrm{Act}, s_0, \mathbf{P})$ *be an MDP as in Setting 2.2. A* subsystem $\mathcal{M}' \subseteq \mathcal{M}$ *is an MDP* $\mathcal{M}' = (S'_{\mathrm{all}}, \mathrm{Act}, s_0, \mathbf{P}')$ *with* $\mathrm{fail}, \mathrm{goal} \in S'_{\mathrm{all}} \subseteq S_{\mathrm{all}}$, $\mathrm{Act}_{\mathcal{M}'}(s) = \mathrm{Act}_{\mathcal{M}}(s)$ *for all* $s \in S'_{\mathrm{all}}$, *and for all* $s, t \in S'_{\mathrm{all}}$ *with* $t \neq \mathrm{fail}$ *and* $\alpha \in \mathrm{Act}$ *we have*

$$\mathbf{P}'(s, \alpha, t) > 0 \Longrightarrow \mathbf{P}'(s, \alpha, t) = \mathbf{P}(s, \alpha, t).$$

*We say that the states* $S_{\mathrm{all}} \setminus S'_{\mathrm{all}}$ *and the transitions* $(s, \alpha, t)$ *with* $\mathbf{P}(s, \alpha, t) > 0$ *and* $\mathbf{P}'(s, \alpha, t) = 0$ *have been* deleted *in* $\mathcal{M}'$. *A* witness *for* $\mathbf{Pr}^{\min/\max}_{\mathcal{M},s_0}(\Diamond\,\mathrm{goal}) \gtrsim \lambda$ *is a subsystem* $\mathcal{M}' \subseteq \mathcal{M}$ *such that* $\mathbf{Pr}^{\min/\max}_{\mathcal{M}',s_0}(\Diamond\,\mathrm{goal}) \gtrsim \lambda$.

*Remark 4.2.* The condition $\mathrm{Act}_{\mathcal{M}'}(s) = \mathrm{Act}_{\mathcal{M}}(s)$ ensures that the probability of a deleted transition $(s, \alpha, t)$ is added to $(s, \alpha, \mathrm{fail})$. This is essential for witnesses for $\mathbf{Pr}^{\min}_{\mathcal{M},s_0}(\Diamond\,\mathrm{goal}) \gtrsim \lambda$ as one could otherwise remove entire actions causing low probabilities and obtain greater $\mathbf{Pr}^{\min}$ in $\mathcal{M}'$ than in $\mathcal{M}$ as a result. For witnesses of $\mathbf{Pr}^{\max}_{\mathcal{M}',s_0}(\Diamond\,\mathrm{goal}) \gtrsim \lambda$ one could delete this condition, thus leading to the notion of [77, 78].

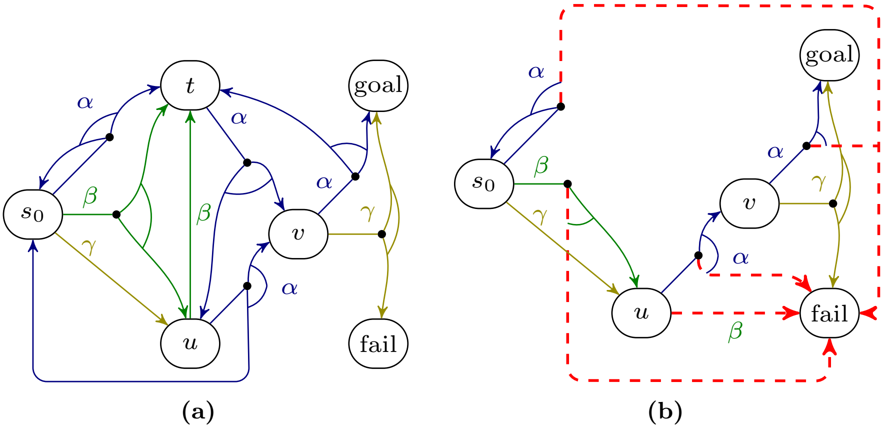

**Fig. 1:** An MDP (with omitted probabilities (a)) and a subsystem (b), where redirected transitions are dashed.

*Example 4.3.* Figure 1a depicts an MDP and Figure 1b indicates the subsystem that is obtained by deleting the state $t$ and additionally the transition $(u, \alpha, s_0)$.

The following lemma ensures that we can use the subsystems as witnesses for both $\mathbf{Pr}^{\max}_{\mathcal{M},s_0}(\Diamond\,\text{goal}) \gtrsim \lambda$ and $\mathbf{Pr}^{\min}_{\mathcal{M},s_0}(\Diamond\,\text{goal}) \gtrsim \lambda$.

**Lemma 4.4.** *Let $\mathcal{M}$ be an MDP as in Setting 2.2 and $\mathcal{M}' \subseteq \mathcal{M}$. Then:*

$$\mathbf{Pr}^{\min}_{\mathcal{M}',s_0}(\Diamond\,\text{goal}) \leq \mathbf{Pr}^{\min}_{\mathcal{M},s_0}(\Diamond\,\text{goal}) \quad \textit{and} \quad \mathbf{Pr}^{\max}_{\mathcal{M}',s_0}(\Diamond\,\text{goal}) \leq \mathbf{Pr}^{\max}_{\mathcal{M},s_0}(\Diamond\,\text{goal})$$

We consider the following notions of minimality for subsystems:

(1) *State-minimality:* $|S'_{\text{all}}|$ is minimal.
(2) *Transition-minimality:* The number of transitions, i.e. triples $(s, \alpha, t)$ satisfying $\mathbf{P}'(s, \alpha, t) > 0$, is minimal;
(3) *Size-minimality:* The sum of states and transitions is minimal.

Depending on the situation, one notion might be more suitable than the others. However, in the full version [42] we show that finding transition-minimal (respectively, size-minimal) witnesses can be reduced to finding state-minimal witnesses with a linear (respectively, quadratic) blow-up. We will therefore restrict ourselves to state-minimality for the rest of this paper.

**NP-completeness of finding minimal witnesses for DTMCs.** In this section we determine the computational complexity of the *witness problem*: Given a DTMC $\mathcal{M}$, a positive integer $k$, and a rational number $\lambda \in [0, 1]$, decide whether there exists a witness $\mathcal{M}' \subseteq \mathcal{M}$ for $\Pr_{\mathcal{M},s_0}(\Diamond\,\text{goal}) \geq \lambda$ with at most $k$ states. The corresponding problem for MDPs is known to be NP-complete [24, 78][1]. In this section we show that the witness problem is already

[1] Although the framework in [24] considers a richer logic, the hardness proof uses only probabilistic reachability formulas such as the ones we consider.

NP-complete for acyclic DTMCs, where acyclicity means that the underlying graph with $V = S$ and $E = \{(s,t) \in S \times S \mid \mathbf{P}(s,t) > 0\}$ is acyclic (as before, we take $S = S_{\text{all}} \setminus \{\text{goal}, \text{fail}\}$). This answers a conjecture of [77] in the affirmative and also shows NP-completeness of finding minimal witnesses for $\mathbf{Pr}^{\min}_{\mathcal{M},s_0}(\lozenge\, \text{goal}) \geq \lambda$.

**Theorem 4.5.** *The witness problem is NP-complete for acyclic DTMCs.*

*Proof (Sketch).* An NP-algorithm for the witness problem is given by guessing a set of states of size $k$ and verifying in polynomial time that the corresponding subsystem satisfies $\Pr_{\mathcal{M}',s_0}(\lozenge\, \text{goal}) \geq \lambda$.

For hardness, we give a reduction from the *clique problem*, which is among Karp's 21 NP-complete problems [54]. The idea is the following: Given an instance of the clique problem with graph $G = (V, E)$ and integer $k$, construct an acyclic Markov chain $\mathcal{M}$ with states $S = \{s_0\} \cup V \cup E \cup \{\text{goal}, \text{fail}\}$ and edges from each vertex $v \in V$ to all edges to which it is incident. Then the existence of a $k$-clique can be reduced to the existence of a "saturated" subsystem in $\mathcal{M}$ with $k$ states in $V$. To check whether the subsystem is saturated, we require it to have more probability than a certain threshold, which depends on $k$ and $|V|$. Details can be found in the full version [42].

*Remark 4.6.* NP-completeness of transition-minimal and size-minimal versions of the witness problem for acyclic DTMCs follows along the same lines, where only the sizes and thresholds for the subsystems need to be adapted.

However, DTMCs whose underlying graph is a tree permit an efficient algorithm for computing minimal witnesses (for the proof see the full version [42]).

**Proposition 4.7.** *Minimal witnesses in tree-shaped DTMCs can be computed in polynomial time.*

*Proof (Sketch).* The algorithm first transforms the DTMC at hand into a binary (tree-shaped) DTMC, and then works bottom up by storing for each state the highest probability that can be obtained with a subsystem of size $k$, for all $k$ up to the size of the subtree.

## 5 Relating Farkas certificates and minimal witnesses

In this section we establish a strong connection between Farkas certificates on the one hand and witnesses for probabilistic reachability constraints on the other hand. We first note that the set of Farkas certificates for non-strict lower bounds forms a polytope, i.e., a bounded polyhedron.

**Lemma 5.1 (Polytopes of Farkas certificates).** *Let $\mathcal{M} = (S_{\text{all}}, \text{Act}, s_0, \mathbf{P})$ be an MDP as in Setting 2.2 and consider $\mathbf{A} \in \mathbb{R}^{\mathcal{M} \times S}$ and $\mathbf{b} \in \mathbb{R}^S$ introduced there. Then for every $\lambda \in [0,1]$ the polyhedra*

$$\mathcal{P}^{\min}(\lambda) = \{\mathbf{z} \in \mathbb{R}^S \mid \mathbf{Az} \leq \mathbf{b} \wedge \mathbf{z}(s_0) \geq \lambda\}$$

$$\mathcal{P}^{\max}(\lambda) = \{\mathbf{y} \in \mathbb{R}^{\mathcal{M}} \mid \mathbf{y} \geq 0 \wedge \mathbf{yA} \leq \delta_{s_0} \wedge \mathbf{yb} \geq \lambda\}$$

*are both polytopes, called* the polytopes of Farkas certificates.

*Remark 5.2.* For any vector $\mathbf{v} \in \mathbb{R}^n$ the support is defined as $\mathrm{supp}(\mathbf{v}) = \{i \in \{1, ..., n\} \mid \mathbf{v}_i > 0\}$, and analogously for the vector spaces $\mathbb{R}^S$ and $\mathbb{R}^{\mathcal{M}}$. As our connection between subsystems of $\mathcal{M}$ and points in $\mathcal{P}^{\min}(\lambda)$ is based on taking the support, we restrict our attention to the subpolytope $\mathcal{P}^{\min}_{\geq 0}(\lambda) = \mathcal{P}^{\min}(\lambda) \cap \mathbb{R}^S_{\geq 0}$.

**Notation 5.3.** Given an MDP $\mathcal{M} = (S_{\mathrm{all}}, \mathrm{Act}, s_0, \mathbf{P})$ as in Setting 2.2 and a subset $R \subseteq \mathcal{M}$, where $\mathcal{M}$ also denotes the state-action pairs (compare with Section 2). We let $\mathcal{M}_R = (S'_{\mathrm{all}}, \mathrm{Act}, s_0, \mathbf{P}')$ be the subsystem where, roughly speaking, the state-action pairs in $R$ *remain*. More precisely, let

$$S'_{\mathrm{all}} = \{s \in S \mid \exists \alpha \in \mathrm{Act}\,.\,(s, \alpha) \in R\} \cup \{\mathrm{goal}, \mathrm{fail}\}$$

$$\mathbf{P}'(s, \alpha, t) = \begin{cases} \mathbf{P}(s, \alpha, t) & \text{if } (s, \alpha) \in R \text{ and } t \in S'_{\mathrm{all}} \setminus \{\mathrm{fail}\} \\ 1 - \sum_{t \in S'_{\mathrm{all}} \setminus \{\mathrm{fail}\}} \mathbf{P}(s, \alpha, t) & \text{if } (s, \alpha) \in R \text{ and } t = \mathrm{fail} \\ 1 & \text{if } (s, \alpha) \notin R, \alpha \in \mathrm{Act}(s) \text{ and } t = \mathrm{fail} \\ 0 & \text{else} \end{cases}$$

For $R \subseteq S$ we set $\mathcal{M}_R = \mathcal{M}_{R'}$ for $R' = \bigcup_{s \in R} \{s\} \times \mathrm{Act}(s)$.

**Theorem 5.4 (Farkas certificates yield witnesses).** *Let $\mathcal{M}$ be an MDP as in Setting 2.2 and $\lambda \in [0, 1]$. Then for a set $R \subseteq S$ the following statements are equivalent:*

*(1) The subsystem $\mathcal{M}_R$ is a witness for $\mathbf{Pr}^{\min}_{\mathcal{M}, s_0}(\lozenge\,\mathrm{goal}) \geq \lambda$.*
*(2) There is a point $\mathbf{p}$ in $\mathcal{P}^{\min}_{\geq 0}(\lambda)$ such that $\mathrm{supp}(\mathbf{p}) \subseteq R$.*
*(3) There is a vertex $\mathbf{v}$ of $\mathcal{P}^{\min}_{\geq 0}(\lambda)$ such that $\mathrm{supp}(\mathbf{v}) \subseteq R$.*

*Moreover, for a set $R \subseteq \mathcal{M}$ the following statements are equivalent:*

*(a) The subsystem $\mathcal{M}_R$ is a witness for $\mathbf{Pr}^{\max}_{\mathcal{M}, s_0}(\lozenge\,\mathrm{goal}) \geq \lambda$.*
*(b) There is a point $\mathbf{p}$ in $\mathcal{P}^{\max}(\lambda)$ such that $\mathrm{supp}(\mathbf{p}) \subseteq R$.*
*(c) There is a vertex $\mathbf{v}$ of $\mathcal{P}^{\max}(\lambda)$ such that $\mathrm{supp}(\mathbf{v}) \subseteq R$.*

One consequence of Theorem 5.4 is that every MD-scheduler $\mathfrak{S}$ with $\mathrm{Pr}^{\mathfrak{S}}_{s_0}(\lozenge\,\mathrm{goal}) \geq \lambda$ corresponds to a point in $\mathcal{P}^{\max}(\lambda)$, i.e. to a certificate for $\mathbf{Pr}^{\max}_{\mathcal{M}, s_0}(\lozenge\,\mathrm{goal}) \geq \lambda$.

**Corollary 5.5 (Detecting minimal witnesses by vertices of $\mathcal{P}$).** *Let $\mathcal{M} = (S_{\mathrm{all}}, \mathrm{Act}, s_0, \mathbf{P})$ be an MDP as in Setting 2.2 and $\lambda \in [0, 1]$. Then a vertex $\mathbf{v}$ of $\mathcal{P}^{\min}_{\geq 0}(\lambda)$ has a maximal number of zeros among all vertices of $\mathcal{P}^{\min}_{\geq 0}(\lambda)$ if and only if $\mathcal{M}_{\mathrm{supp}(\mathbf{v})}$ is a minimal witness for $\mathbf{Pr}^{\min}_{s_0}(\lozenge\,\mathrm{goal}) \geq \lambda$.*

*Dually, a vertex $\mathbf{v}$ of $\mathcal{P}^{\max}(\lambda)$ has a maximal number of zeros among all vertices of $\mathcal{P}^{\max}(\lambda)$ if and only if all of the following hold:*

*(1) $\mathcal{M}_{\mathrm{supp}(\mathbf{v})} = (S'_{\mathrm{all}}, \mathrm{Act}, s_0, \mathbf{P}')$ is a minimal witness for $\mathbf{Pr}^{\max}_{s_0}(\lozenge\,\mathrm{goal}) \geq \lambda$,*
*(2) for every $s \in S'$ there is precisely one $\alpha \in \mathrm{Act}(s)$ with $(s, \alpha) \in \mathrm{supp}(\mathbf{v})$,*
*(3) the corresponding map $\mathfrak{S}\colon S' \to \mathrm{Act}$ is an MD-scheduler on $\mathcal{M}_{\mathrm{supp}(\mathbf{v})}$ with $\mathrm{Pr}^{\mathfrak{S}}_{s_0}(\lozenge\,\mathrm{goal}) \geq \lambda$.*

## 6 Computing witnessing subsystems

In this section we use the results of Section 5 to derive two algorithms for the computation of minimal witnesses for reachability constraints in MDPs. As the problem is NP-hard, we also present a heuristic approach aimed at computing small witnessing subsystems.

**Vertex enumeration.** Corollary 5.5 gives rise to the following approach of computing minimal witnessing subsystems: enumerate all vertices in the corresponding polytope and choose one with a maximal amount of zeros. Vertex enumeration of polytopes has been studied extensively [11, 12, 14, 21, 22, 35, 36, 40, 41, 63, 68] and has been shown to be computationally hard [55, Corollary 2].

First experiments that we have conducted with the `SageMath`[2] toolkit which supports vertex enumeration have not scaled well in the dimension, which in our case is the number of states in the original system. Also, we found no tool support for vertex enumeration that is able to handle sparse matrices, which is essential for bigger benchmarks.

**Mixed integer linear programming.** An approach that computes minimal witnesses to the threshold problem $\mathbf{Pr}^{\max}_{s_0}(\lozenge\, \text{goal}) \geq \lambda$ using mixed integer linear programs (MILP) was presented in [77, 78]. Using the following lemma, we can derive MILP formulations from our polytope formulations.

**Lemma 6.1.** *Let $\mathcal{P} = \{\mathbf{x} \mid \mathbf{A}\mathbf{x} \leq \mathbf{b}, \mathbf{x} \geq 0\} \subseteq \mathbb{R}^n$ be a polytope and $K \geq 0$ be such that for all $\mathbf{p} \in \mathcal{P}$ and $1 \leq i \leq n$ we have $\mathbf{p}(i) \leq K$. Consider the MILP*

$$\min \sum_{1 \leq i \leq n} \boldsymbol{\sigma}(i) \quad s.t. \quad \mathbf{x} \in \mathcal{P}, \quad \mathbf{x} \leq K \cdot \boldsymbol{\sigma}, \quad \boldsymbol{\sigma}(i) \in \{0, 1\}$$

*Then a vector $(\boldsymbol{\sigma}, \mathbf{x})$ is an optimal solution of this MILP if and only if $\mathbf{x}$ is a point in $\mathcal{P}$ with a maximal number of zeros.*

For $\mathcal{P}^{\min}_{\geq 0}(\lambda)$ we can use Lemma 3.1 to derive that $K = 1$ is a viable bound. By invoking again Corollary 5.5, this means that a solution $(\mathbf{z}, \boldsymbol{\sigma})$ of the MILP

$$\min \sum_{s \in S} \boldsymbol{\sigma}(s) \;\text{ s.t. }\; \mathbf{z} \in \mathcal{P}^{\min}_{\geq 0}(\lambda), \quad \mathbf{z} \leq \boldsymbol{\sigma}, \quad \boldsymbol{\sigma}(i) \in \{0, 1\}$$

encodes a minimal witnessing subsystem in the integral variables $\boldsymbol{\sigma}$. This MILP was used in [77, 78] for the computation of minimal witnessing subsystems of DTMCs .

An upper bound $K$ as in Lemma 6.1 for $\mathcal{P}^{\max}(\lambda)$ can be found in polynomial time by taking the objective value of an optimal solution to the LP

$$\max \sum_{(s,\alpha) \in \mathcal{M}} \mathbf{y}(s, \alpha) \;\text{ s.t. }\; \mathbf{y} \in \mathcal{P}^{\max}(\lambda)$$

[2] http://www.sagemath.org/

*Remark 6.2.* To compute minimal witnesses for $\mathbf{Pr}^{\max}_{s_0}(\lozenge\,\mathrm{goal}) \geq \lambda$, [77, 78] (witnesses for $\mathbf{Pr}^{\min}_{s_0}(\lozenge\,\mathrm{goal}) \geq \lambda$ were not considered) propose the MILP with objective: min $\sum_{(s,\alpha)\in\mathcal{M}} \boldsymbol{\sigma}(s,\alpha)$, subject to the conditions

$$\forall (s,\alpha) \in \mathcal{M}. \quad \mathbf{z}(s) \leq 1 - \boldsymbol{\sigma}(s,\alpha) + \sum_{s'\in S} \mathbf{P}(s,\alpha,s') \cdot \mathbf{z}(s') + \mathbf{b}(s) \tag{6.1}$$

$$\forall s \in S. \quad \mathbf{z}(s) \leq \sum_{\alpha\in\mathrm{Act}(s)} \boldsymbol{\sigma}(s,\alpha), \quad \mathbf{z}(s_0) \geq \lambda \tag{6.2}$$

where $\boldsymbol{\sigma}(s,\alpha)$ are binary integer variables. It was implemented in the tool `ltlsubsys`. The idea is to directly encode a scheduler in the set of equations $\mathbf{Az} \leq \mathbf{b}$ using $\boldsymbol{\sigma}$. In [77, 78] a number of additional redundant constraints are given to guide the search. In contrast to [77, 78] we do not need to handle so-called *problematic states*, as our precondition $\mathbf{Pr}^{\min}_{s}(\lozenge(\mathrm{goal} \vee \mathrm{fail})) > 0$ guarantees that no such states exist.

***k*-step quotient sum (QS$_k$) heuristics.** Approximating the maximal number of zeros in a polytope is computationally hard in general [8]. We now derive a heuristic approach for this problem called *quotient sum heuristic* which is based on iteratively solving LPs over the polytope, where the objective function for each iteration depends on an optimal solution of the previous LP. More precisely, we take $\mathbf{o}_1 = (1,\ldots,1)$ and take an optimal solution $\mathrm{QS}_1$ of the LP $\min \mathbf{o}_1 \cdot \mathbf{y}$ s.t. $\mathbf{y} \in \mathcal{P}^{\max}(\lambda)$. Many entries in $\mathrm{QS}_1$ may be small, but still greater than zero. In order to push as many of the small values of $\mathrm{QS}_1$ to zero, we define a new objective function by

$$\mathbf{o}_2(i) = \begin{cases} 1/\,\mathrm{QS}_1(i), & \text{if } \mathrm{QS}_1(i) > 0 \\ C, & \text{if } \mathrm{QS}_1(i) = 0 \end{cases} \tag{6.3}$$

where $C$ is a value that is greater than any value $1/\,\mathrm{QS}_1(i)$. We now take a solution $\mathrm{QS}_2$ of the new LP $\min \mathbf{o}_2 \cdot \mathbf{y}$ s.t. $\mathbf{y} \in \mathcal{P}^{\max}(\lambda)$ and form the next objective function $\mathbf{o}_3$ as in (6.3). Inductively this generates a sequence of objective functions $(\mathbf{o}_k)_{k\geq 1}$ and corresponding optimal solutions $(\mathrm{QS}_k)_{k\geq 1}$ in $\mathcal{P}^{\max/\min}(\lambda)$. By Theorem 5.4 we can construct a witnessing subsystem with as many states as the number of non-zero entries in $\mathrm{QS}_k$.

## 7 Experiments

In this section we evaluate our MILP formulations and heuristics on a number of DTMC and MDP benchmarks from the Prism benchmark-suite [58, 59]. We compare our results with the tool Comics [50], which implements heuristic approaches to compute small subsystems for DTMCs. It has two modes: the *local search* extends a given subsystem by short paths that carry much probability, whereas the *global search* searches for the next most probable path from the initial state to goal, and adds it to the subsystem. Both approaches iteratively extend a subsystem until it carries more probability than the given threshold and thus have to compute the probability of the subsystem at each iteration.

All computations were performed on a computer with two Intel E5-2680 8 cores at 2.70 GHz running Linux, with a time bound of 30 minutes, a memory bound of 100 GB and with each benchmark instance having access to 4 cores. For the LP and MILP instances we use the Gurobi solver, version 8.1.1 [43]. The recorded times of our computations include the construction of the LPs/MILPs and are wall clock times. Pre-processing steps, such as collapsing states that cannot reach goal, are not counted in the time consumption. For Comics, we use the time that is reported as counterexample generation time by the tool.

To validate our implementation, we used Prism to verify that the subsystems that we compute indeed satisfy the probability thresholds. We noticed that for a few instances ($< 0.5\%$) Prism reported a deviation of less than $10^{-8}$, which can be explained by the fact that both Prism and the solvers that we use rely on floating-point arithmetic, which is approximate by nature.

Our implementation, together with the models we use and benchmark results can be found at https://github.com/simonjantsch/farkas.

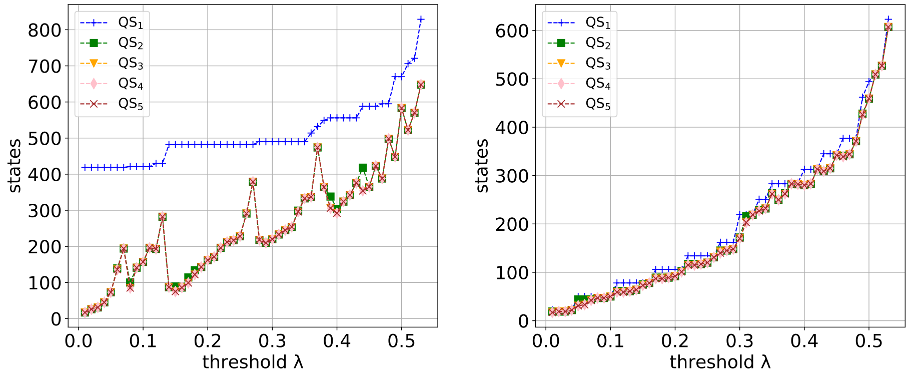

**(a)** QS-heuristic applied to $\mathcal{P}^{\max}(\lambda)$. **(b)** QS-heuristic applied to $\mathcal{P}^{\min}_{\geq 0}(\lambda)$.

**Fig. 2:** crowds-2-8: comparing $\mathrm{QS}_k$ for growing $k$.

**DTMC benchmarks.** As $\mathbf{Pr}^{\max}$ and $\mathbf{Pr}^{\min}$ coincide on DTMCs, we can use the heuristics and exact computations derived from either the $\mathcal{P}^{\max}$ or the $\mathcal{P}^{\min}_{\geq 0}$ polytope for DTMCs (in Comics we use the standard query $\mathrm{Pr}_{s_0}(\Diamond\, \mathrm{goal}) \geq \lambda$). We consider two DTMC benchmarks: a model of the crowds-$N$-$K$ protocol [69, 72] for ensuring anonymous web browsing (with $N$ members and $K$ protocol runs) and a model of the bounded retransmission protocol [34, 47] for file transfers (where brp-$N$-$K$ is the instance with $N$ chunks and $K$ retransmissions).

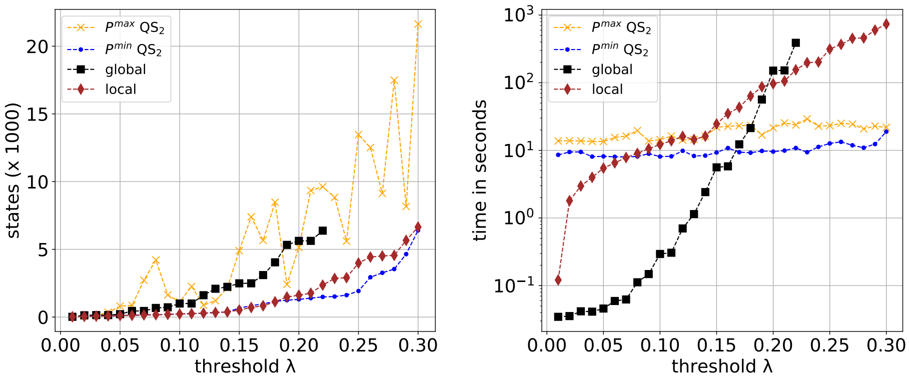

**(a)** crowds-5-8 (46,873 states). Comics-global runs out of memory for $\lambda \geq 0.23$.

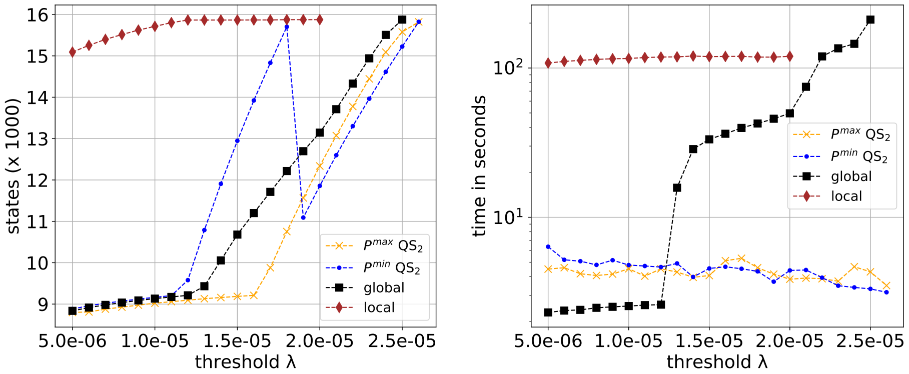

**(b)** brp-512-2 (15,875 states). Comics-local reports an error for $\lambda \geq 2.1 \cdot 10^{-5}$ and Comics-global runs out of memory for $\lambda = 2.6 \cdot 10^{-5}$.

**Fig. 3:** Comparison of heuristic methods on DTMC benchmarks.

Figure 2 shows the effect of increasing the number of iterations of the QS-heuristic for the model crowds-2-8. While the first iteration (taking $QS_2$ instead of $QS_1$) has an impact on the number of states, more iterations do not improve the result significantly. For $QS_1$, the sizes of subsystems increase monotonically with growing $\lambda$. Starting with $QS_2$ the results may, interestingly, have “spikes”: increasing $\lambda$ can lead to smaller subsystems.

Figure 3 shows the results of the $QS_2$-heuristic compared to the two modes of Comics for $\lambda$ that ranges between 0 and the actual reachability probability of the model. A general observation is that the runtime of the QS-heuristic is independent of $\lambda$, whereas both modes of Comics use significantly more time with increasing $\lambda$. The same observation can be done for memory consumption, which stayed below 200 MB for our heuristics. Also, especially for crowds-5-8, one can see that relatively small subsystems are possible even for large $\lambda$. The exact computations via MILPs hit the timeout for almost all instances.

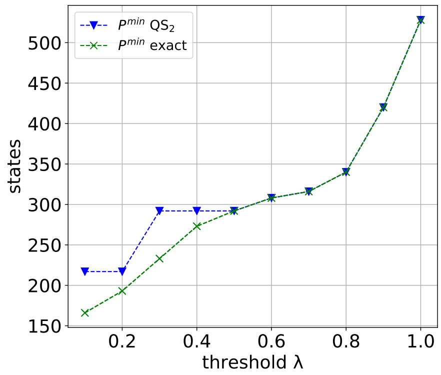

**(a)** Witnesses for $\mathbf{Pr}_{s_0}^{\min}(\lozenge\,\text{goal}) \geq \lambda$.

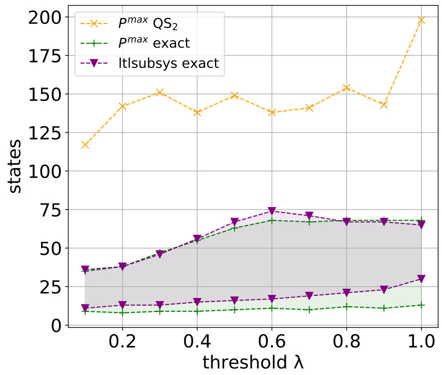

**(b)** Witnesses for $\mathbf{Pr}_{s_0}^{\max}(\lozenge\,\text{goal}) \geq \lambda$.

**Fig. 4:** MDP benchmark: consensus-2-4 (528 states)

In Figure 3 it can be seen that the QS heuristics derived from the two polytopes $\mathcal{P}^{\max}$ and $\mathcal{P}^{\min}_{\geq 0}$ may produce different results. However, for both models one of them gives monotonically growing subsystems and outperforms COMICS. While QS$_2$ applied to $\mathcal{P}^{\min}_{\geq 0}$ performs better on crowds-5-8 (Figure 3a), it is the other way around on brp-512-2 (Figure 3b). In future work we intend to investigate what properties determine which of the two formulations performs better for a given DTMC.

**MDP benchmarks.** We consider two MDP models: the randomized consensus-$N$-$K$ protocol of [10, 60] (with $N$ processes and a bound $K$ on the random walk) and the CSMA-$N$-$K$ protocol for data channels [61] (where $N$ is the number of stations, and $K$ is the maximal backoff count). The results of both heuristic and exact computations can be seen in Figure 4 and Figure 5. Whereas the heuristics all needed less than 5 minutes, all MILP instances ran into the timeout except for the ones in Figure 4a. Whenever a MILP instance could not be solved optimally in 30 minutes, we plot both the found upper and lower bound, with the region in between shaded. It should be noted that the condition $\mathbf{Pr}^{\min}(\lozenge(\text{goal} \vee \text{fail}))$ holds for the instances of these models, and reachability properties, that we consider.

The comparison between the MILP formulation that we derived from $\mathcal{P}^{\max}(\lambda)$ and the one presented in [77, 78] (labeled by `ltlsubsys`, see also Section 6) shows that both compute comparable upper and lower bounds in Figure 4b, whereas `ltlsubsys` found worse upper bounds in Figure 5b. In all instances apart from Figure 4b the corresponding QS$_2$ heuristics performs well and generates subsystems that are as good, or better, than the best upper bounds computed by the MILPs in 30 minutes. As expected, the witnessing subsystems for $\mathbf{Pr}_{s_0}^{\min}(\lozenge\,\text{goal}) \geq \lambda$ tend to the entire state space as $\lambda$ tends to the actual value $\mathbf{Pr}_{s_0}^{\min}(\lozenge\,\text{goal})$ (which is 1 in these two models). However, subsystems for $\mathbf{Pr}_{s_0}^{\max}(\lozenge\,\text{goal}) \geq \lambda$ may be substantially smaller even for large $\lambda$.

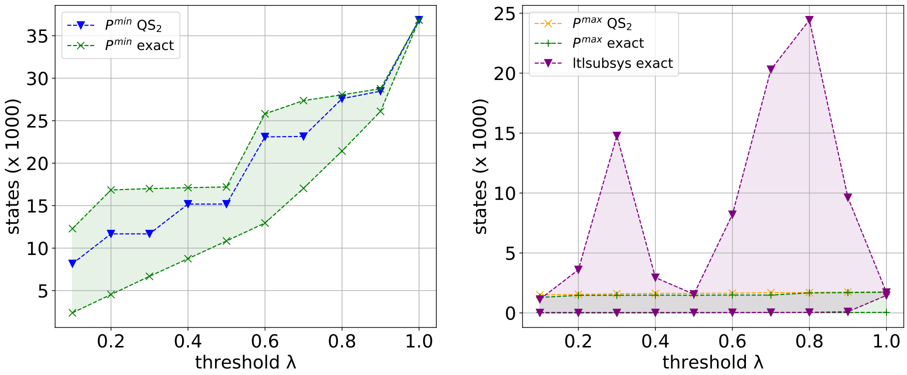

**(a)** Witnesses for $\mathbf{Pr}^{\min}_{s_0}(\lozenge\,\mathrm{goal}) \geq \lambda$. **(b)** Witnesses for $\mathbf{Pr}^{\max}_{s_0}(\lozenge\,\mathrm{goal}) \geq \lambda$.

**Fig. 5:** MDP benchmark: CSMA-3-2 (36,850 states)

## 8 Conclusion

In this paper we brought together two a priori unrelated notions in the context of probabilistic reachability constraints: on the one hand Farkas certificates, which are vectors satisfying certain linear inequalities that we derive using MDP-specific variants of Farkas' Lemma, and on the other hand witnessing subsystems, which provide insight into which parts of the system are essential for the satisfaction of the considered property. This connection reduces the computation of minimal (respectively, small) witnessing subsystems to finding a Farkas certificate with a maximal (respectively, large) number of zeros. Furthermore, it leads to a unified notion of witnessing subsystem for $\mathbf{Pr}^{\max}_{s_0}(\lozenge\,\mathrm{goal}) \geq \lambda$ and $\mathbf{Pr}^{\min}_{s_0}(\lozenge\,\mathrm{goal}) \geq \lambda$.

We showed that the decision version of computing minimal witnessing subsystems is NP-complete for acyclic DTMCs and introduced heuristics for the computation of small witnesses based on Farkas certificates. Experiments of the heuristics exhibited competitive results compared to the approach implemented in Comics and showed that they scale well with the system size and threshold. As expected, computing minimal subsystems using the derived MILP formulations consumed significantly more time than the heuristics and often triggered timeouts. The upper and lower bounds that were computed in the given time by the new MILP formulation for $\mathbf{Pr}^{\max}_{s_0}(\lozenge\,\mathrm{goal}) \geq \lambda$ were comparable to known techniques.

We have considered MDPs in which the probability to reach goal or fail is positive under each scheduler. In future work, we plan to extend our techniques to weaken this assumption. Exploring how vertex enumeration techniques could be adapted to the MDP-specific form of the Farkas polytopes is another interesting line of future work. We also plan to implement a tool for working with Farkas certificates in practice, which encompasses their generation as well as their independent validation.
